# Complete genome sequence of three *Staphylococcus epidermidis* strains isolated from patients with skin diseases in Japan

**DOI:** 10.1128/mra.00179-24

**Published:** 2024-06-20

**Authors:** Yu Kawagishi, Teresia M. Kimeu, Kazunori Murase, Akemi Yoshida, Atsuko Minowa-Nozawa, Takashi Nozawa, Yasuhiro Tsuchido, Taro Noguchi, Miki Nagao, Saeko Nakajima, Ichiro Nakagawa

**Affiliations:** 1 Department of Microbiology, Graduate School of Medicine, Kyoto University, Kyoto, Japan; 2 Frontier Science Research Center, Faculty of Medicine, University of Miyazaki, Miyazaki, Japan; 3 Clinical Laboratory Medicine, Graduate School of Medicine, Kyoto University, Kyoto, Japan; 4 Department of Dermatology, Graduate School of Medicine, Kyoto University, Kyoto, Japan; Loyola University Chicago, Chicago, Illinois, USA

**Keywords:** coagulase-negative staphylococci, antibiotic resistance

## Abstract

*Staphylococcus epidermidis* is a member of the human skin microbiota as a commensal organism but could be an important opportunistic pathogen for immunocompromised individuals. Here, we report the complete genome sequence of three *S*. *epidermidis* strains isolated from patients with skin diseases.

## ANNOUNCEMENT

*Staphylococcus epidermidis*, a coagulase-negative bacterium, is a major bacterial species of human skin microbiota (1, 2). *S. epidermidis* has been thought to maintain skin homeostasis by promoting cutaneous immune responses and preventing opportunistic pathogens from causing disease through colonization resistance (3, 4). On the other hand, some strains of *S. epidermidis* have been reported to be possibly involved in the pathogenesis of common skin diseases (5). In addition, *S. epidermidis* has various antimicrobial resistance genes through mobile genetic elements (6). Genomic studies on *S. epidermidis* have shown the emergence of multidrug-resistance lineages in recent decades and their international spread (7, 8). Here, we report the complete genome sequence of *S. epidermidis* strains, which were isolated from the skin of patients with psoriasis or atopic dermatitis in Japan.

To obtain *S. epidermidis* isolates, skin microbial samples were collected from 4 cm^2^ areas of the skin (back or major trochanter of the femur) using Hydra flock swab soaked in enzymatic lysis buffer (ethics approved number R0743: The Ethics Committee of Kyoto University). The skin swabs were then spread on Trypticase soy agar plate, and single colonies were identified by matrix-assisted laser desorption/ionization time-of-flight mass spectrometry (MALDI-TOF MS) (9). The *S. epidermidis* strains were incubated for 15 h at 37°C in Trypticase soy medium. The bacterial cells were lysed with lysostaphin and lysozyme, and then the genomic DNA was extracted using the phenol-chloroform extraction method (10). Extracted genomic DNA was sequenced in short- and long-read sequencing platforms.

A short-read library was prepared using an Illumina DNA prep kit, and paired-end reads were generated using a MiSeq reagent kit v3 (600 cycles, 2 × 300 bp paired-end reads) on the MiSeq platform (Illumina). A long-read library was prepared from unsheared genomic DNA using the Rapid Barcoding kit 24 V14 (SQK-RBK114.24) and sequenced with an R10.4.1 flow cell (FLO-MIN114) on a MinION device (Oxford Nanopore Technologies). The Illumina data were preprocessed using Fastp v0.36 (11) to remove the adapter and low-quality sequences. For base calling and demultiplexing of Oxford Nanopore Technologies (ONT) data, Guppy v7.1.4 with high accuracy mode was used, and the reads were further processed using Porechop v0.2.4 (12) and Filtlong v0.2.1 (https://github.com/rrwick/Filtlong). High-quality long reads were assembled using Flye v2.9.2 (13). We further run Plassembler v1.5.0 (14) with short and long reads, and small circularity contigs annotated with PLSDB (release 2023_11_03_v2) and not mapping on their representative chromosome were identified as reliable plasmids. The complete sequence was polished using Medaka v1.8.1 (https://github.com/nanoporetech/medaka) and Polypolish v0.5.0 (15) for error correction, and the polished sequences were rotated using the fixstart task in Circlator v1.5.5 (16) to fix the start position. Genome annotation was performed by DFAST pipeline v1.2.18 (17). Antimicrobial resistance genes were identified using AMRFinderPlus (18). Unless otherwise noted, default parameters were used for all software tools. The Illumina and ONT read stats, assembly statistics, general genome information, and relevant characteristics are summarized in Table 1.

**TABLE 1:** Assembly statistics and general genome information of *S. epidermidis* strains

		Data for *S. epidermidis* strains	
Statistic or characteristic	KUHPSE03	KUHPSE08	KUHPSE09
Strain information			
Origin	Skin	Skin	Skin
Clinical symptom	Psoriasis	Atopic dermatitis	Atopic dermatitis
Yr of isolation	2018	2018	2018
Assembly and genome statistics			
No. of reads			
MinION	177,377	170,595	64,509
MiSeq	595,335	507,629	706,023
Total no. of bases			
MinION	1,021,112,808	821,291,138	347,251,802
MinION (N50)	8,734	7,675	8,019
MiSeq	321,775,579	275,262,057	377,666,110
Coverage (×)	545.13	453.19	294.23
Accession no.	DRR520170 (Illumina) DRR520173 (Nanopore)	DRR520171 (Illumina) DRR520174 (Nanopore)	DRR520172 (Illumina) DRR520175 (Nanopore)
Chromosome description			
Genome size (bp)	2,463,422	2,419,652	2,463,760
G + C content (%)	32.2	32.3	32.2
No. of CDSs^*b*^	2,269	2,260	2,386
No. of rRNAs	19	19	19
No. of tRNAs	60	60	60
AMR genes^*a*^	*fosB*, *blaZ*, and *blaI*	*fosB, rpsJ_Y58D aac(6')-Ie/aph(2'')-Ia*	*fosB*, *blaZ*, and *blaI*
Accession no.	AP029227	AP029231	AP029237
Plasmid description			
No. of plasmids	3	5	5
Genome size (bp)	47,894, 9,892, and 9,111	34,943, 26,512, 20,885, 4,101, and 2,393	8,372, 4,524, 3,189, 2,268, and 1,279
G + C content (%)	27.82, 28.1, and 30.53	29.06, 31.08, 29.45, 33.46, and 30.71	30.64, 27.34, 28.97, 30.64, and 34.25
No. of CDSs	52 13 9	38 27 21 4 2	11 5 4 2 1
AMR genes	Not detected	*aadD1* (pKUHPSE08-1) *mecA* (pKUHPSE08-2) *mecR1* (pKUHPSE08-2) *blaZ* (pKUHPSE08-3) *blaR1* (pKUHPSE08-3) *blaI* (pKUHPSE08-3) *tet(K)* (pKUHPSE08-3) *erm(C)* (pKUHPSE08-5)	*qacC* (pKUHPSE09-4)
Accession no.	AP029228 AP029229 AP029230	AP029232 AP029233 AP029234 AP029235 AP029236	AP029238 AP029239 AP029240 AP029241 AP029242

^*a*^ Antimicrobial resistance (AMR) genes were identified by AMRFinderPlus.

^*b*^ Coding sequences.

## Data Availability

The complete genome sequences of three *S. epidermidis* strains and the relevant raw sequence data in this study have been deposited in DDBJ/EMBL/GenBank under the accession numbers in Table 1.
